# Using web-based familial risk information for diabetes prevention: a randomized controlled trial

**DOI:** 10.1186/1471-2458-13-485

**Published:** 2013-05-17

**Authors:** Miranda Wijdenes, Lidewij Henneman, Nadeem Qureshi, Piet J Kostense, Martina C Cornel, Danielle RM Timmermans

**Affiliations:** 1 Department of Public and Occupational Health, EMGO Institute for Health and Care Research, VU University Medical Center, Amsterdam, The Netherlands; 2 Department of Clinical Genetics, section Community Genetics, EMGO Institute for Health and Care Research, VU University Medical Center, Amsterdam, The Netherlands; 3 Division of Primary Care, University of Nottingham, Nottingham, United Kingdom; 4 Department of Epidemiology and Biostatistics, VU University Medical Center, Amsterdam, The Netherlands

**Keywords:** Family history, Type 2 diabetes, Prevention, Common chronic diseases, Risk assessment

## Abstract

**Background:**

It has been suggested that family history information may be effective in motivating people to adopt health promoting behaviour. The aim was to determine if diabetic familial risk information by using a web-based tool leads to improved self-reported risk-reducing behaviour among individuals with a diabetic family history, without causing false reassurance among those without a family history.

**Methods:**

An online sample of 1,174 healthy adults aged 35–65 years with a BMI ≥ 25 was randomized into two groups receiving an online diabetes risk assessment. Both arms received general tailored diabetes prevention information, whilst the intervention arm also received familial risk information after completing a detailed family history questionnaire. Separate analysis was performed for four groups (family history group: 286 control versus 288 intervention group; no family history: 269 control versus 266 intervention group). Primary outcomes were self-reported behavioural outcomes: fat intake, physical activity, and attitudes towards diabetes testing. Secondary outcomes were illness and risk perceptions.

**Results:**

For individuals at familial risk there was no overall intervention effect on risk-reducing behaviour after three months, except for a decrease in self-reported saturated fat intake among low-educated individuals (Beta (*b*) -1.01, 95% CI −2.01 to 0.00). Familial risk information resulted in a decrease of diabetes risk worries (*b −*0.21, -0.40 to −0.03). For individuals without family history no effect was found on risk-reducing behaviour and perceived risk. A detailed family history assessment resulted in a greater percentage of individuals reporting a familial risk for diabetes compared to a simple enquiry.

**Conclusions:**

Web-based familial risk information reduced worry related to diabetes risk and decreased saturated fat intake of those at greatest need of preventative care. However, the intervention was not effective for the total study population on improving risk-reducing behaviour. The emphasis on familial risk does not seem to result in false reassurance among individuals without family history. Additionally, a detailed family history questionnaire identifies more individuals at familial risk than a simple enquiry.

**Trial registration:**

NTR1938

## Background

Type 2 diabetes is increasingly common due to lifestyle factors (physical inactivity, unhealthy diet) [1]. There is convincing evidence from intervention studies in high-risk groups (e.g. overweight people) that weight loss, healthy diet and physical activity can delay or even prevent the onset of diabetes [2]. However, current behavioural programs aimed at diabetes prevention that use general health messages have limited effect [3]. Besides lifestyle factors, family history is an important and independent risk factor for diabetes [4]. Being at familial risk reflects the consequences of genetic predisposition, shared environment and common behaviours. Family history may be used to identify individuals at risk for diabetes and to influence early detection [5]. Besides, it has been suggested that family history information can be used to personalize health messages for individuals at risk, which may be more effective in motivating them to adopt a healthy lifestyle than general health messages [6]. Individuals with a diabetes family history have difficulty understanding the complex interaction between genetic and behavioural causes of diabetes and have limited concerns about getting the disease [7-9], which confines their motivation to adopt health promoting behaviour. Familial risk information resulted in increase diabetes risk perception among those who underestimated their risk [10].

Theories of health behaviour show that risk perception (threat appraisal) [11], as well as illness perceptions, such as causal beliefs and personal control over the risk [12], are important factors associated with motivation to engage in preventive behaviours. Since individual’s perceived risk of disease does not correspond to their actual risk, interventions need to be developed to reduce the mismatch, and to improve awareness of the multifactorial nature of diabetes [7,9]. The latter includes the relevance of a familial risk. Although there has been concern that emphasizing family history may lead to adverse psychological effects, there is no evidence that informing individuals about their familial risk might cause psychological harm [13-15] or leads to a decrease of perceived personal control over the risk (fatalism) [16].

A recent study showed that individuals who received diabetic familial risk in a face-to-face consultation reported higher engagement in risk-reducing behaviours [14]. However, if family history information is used as a public health strategy, then it is necessary to develop a tool that is simple, easily applied, and adaptable to different settings [5,17]. Computer tailoring is seen as an effective way to mimic interpersonal contact, since personalized feedback is provided by means of electronic questions [18]. The advantage of computer tailored information is the possibility to reach a larger number of individuals. A consequence of exploring familial risk in the general population is that individuals with no family history will also be highlighted. This could be problematic since individuals without a diabetes family history might erroneously believe that having no family history indicates that they are not at risk of diabetes, in other words, lead to false reassurance [19]. This belief may lead to unintentional adverse effects, such as reduced motivation to change behaviour, justification of unhealthy behaviour, and delayed seeking of medical advice [20]. Among individuals without a family history the question of false reassurance needs further exploration. In the literature, there is no clear operational definition of false reassurance. In this study it is represented by reduced risk perception and as a consequence less risk-reducing behaviour, as has been done in other studies [21,22].

The aim of this study was to determine the effect of tailored web-based diabetic familial risk information on risk-reducing behaviour and perceptions of individuals. Research questions are:

1. What is the impact of familial risk information on risk-reducing behaviour and illness and risk perceptions of individuals **with** a diabetes family history?

2. Does an intervention that emphasizes familial risk information result in false reassurance among individuals **without** a diabetes family history?

## Methods

The Preventing Diabetes Controlled Trial (PreDiCT) was registered at the Dutch Trial Register (NTR1938) and approved by the VU University Medical Center Ethical Committee.

### Participants and procedure

Study participants were recruited from an independent certified research agency. The research agency provided credit points that could be redeemed for gift cards to encourage participation.

**Figure 1:**
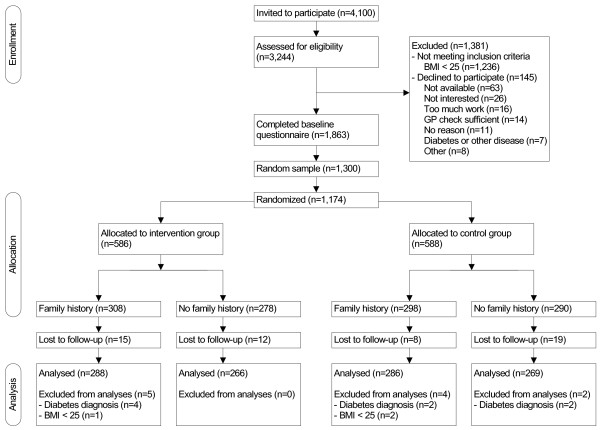
CONSORT flow diagram of the study.

People with one (or more) first-degree relative with diabetes (23%) were identified using a single-item family history question, a month prior to recruitment for the study (April 2009), as part of a larger online general survey of the research agency sampling frame of 88,568. To ensure the trial was adequately powered, 4,100 people were recruited (see sample size section). A random sample of 2,900 individuals with a diabetes family history and 1,200 individuals without a family history respectively were drawn by block randomization. Participants were not aware of being selected because of their familial risk. The study procedure, including the selection and enrolment of participants, is shown in the flow diagram in Figure 1. Included were healthy individuals from the general population aged 35 to 65 years with a Body Mass Index (BMI, kg/m^2^) ≥ 25. Exclusion criteria were being diagnosed with diabetes (type 1 or 2), unable to read and complete questionnaires in Dutch, and being Hindustani, Turkish, Creolish, or Moroccan (because these populations have a higher than average risk of getting diabetes). From the 4,100 individuals invited, 3,244 (79%) people responded to the study invitation per Email and were assessed for inclusion. All participants gave informed consent for participation in the study. Thirty-eight percent (1,236) of individuals with a BMI < 25 were excluded and 145 individuals declined to participate. The remaining 1,863 completed the online baseline study questionnaire (May 2009). From these 1,863, a random sample of 1,300 participants (the number needed based on the power calculation) was invited to complete the intervention (June 2009), of which 1,174 (90%) agreed to participate. From the 1,174 sample, participants were randomized into parallel control and intervention arms by means of a concealed computer-generated list of random numbers.

### Intervention

The design of the web-based information for the intervention arm was based on the results of earlier studies [7,14]. Both groups received general tailored diabetes prevention information, whilst the intervention group also received information based on familial risk (See Additional file 1: Table S1). All participants were informed that the study was to determine the best way to advise people about their diabetes risk and were thus blinded for study groups.

#### *General web-based information* (control group)

**Figure 2:**
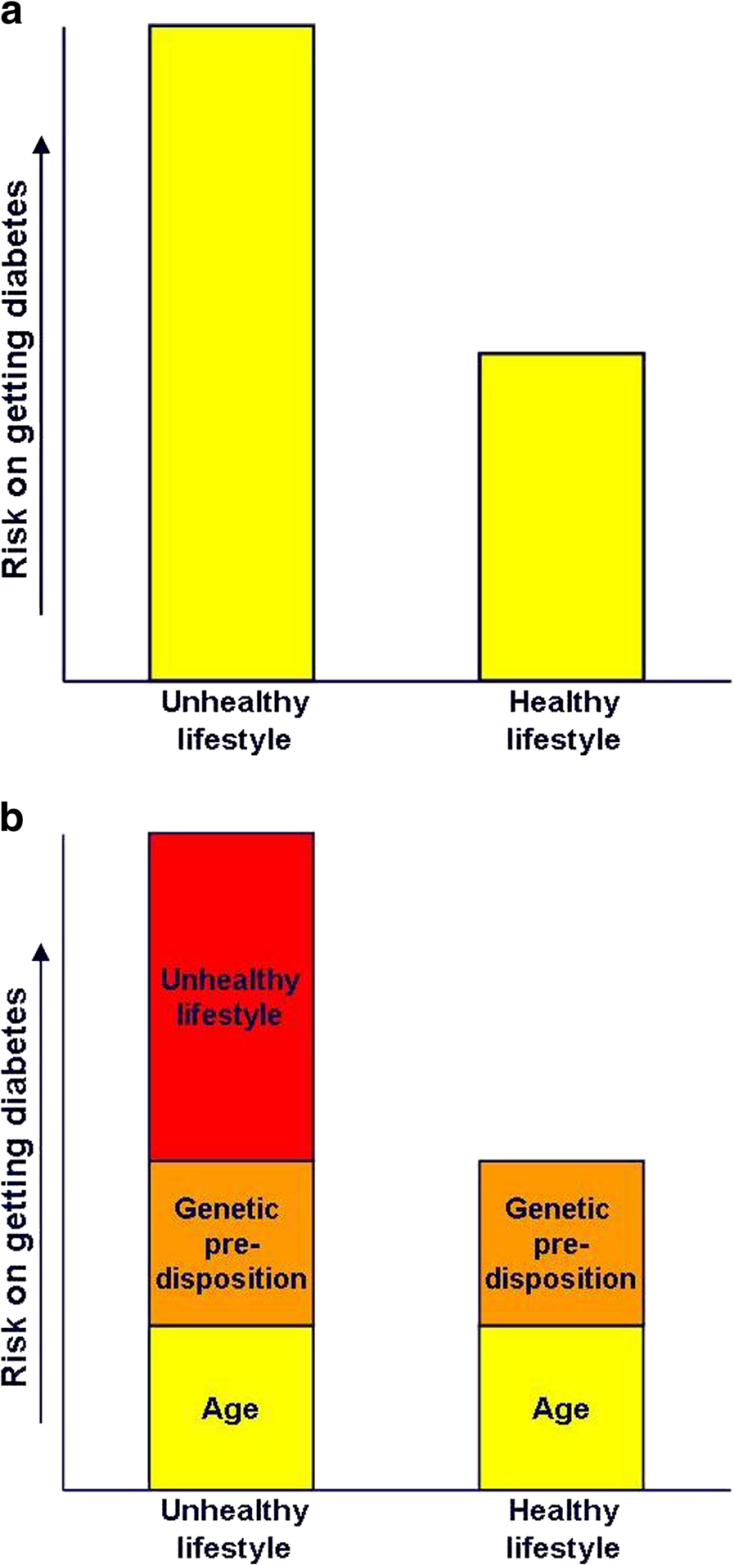
Graphical bar chart presented to the participants in the (a.) control group and (b.) intervention group.

Participants received general information about type 2 diabetes, consisting of a simplified explanation of the metabolic disorder, the diabetes consequences, and main risk factors (not including family history). The effectiveness of preventive options were explained in a bar chart (Figure 2a), indicating that they can reduce their risk by half by adopting a healthy lifestyle.

Diabetes risk was assessed using a Diabetes Risk Test [23] validated for the Dutch population. Family history in this test was assessed by a simple enquiry: ‘Does diabetes occur within your family? 1) no; 2) yes, with my grandfather, grandmother, uncle, aunt, nephew, niece; 3) yes, with my father, mother, brother, sister, or child’.

Diabetes Risk Test results were categorized in three risk strata (2 in 100, 10 in 100 and 20 in 100) that referred to their risk of getting diabetes within the next five years. Each participant received an individual risk based on the risk test, supported by risk-reducing preventive measures tailored to the three risk strata.

After completing the post-test study questionnaire, all participants were invited to visit an evidence based computer-tailored lifestyle modification tool advising on approaches to reduce saturated fat intake and improve physical activity (http://www.leefgezondcoach.nl) [24]. This lifestyle information was not part of the web-based intervention, but was included to give participants the option to reduce their diabetes risk.

#### *Intervention web-based information* (intervention group)

As well as general information, described above, the intervention comprised:

Advice that familial risk increases with the number and kinship of affected relatives. Further, the multifactorial character of type 2 diabetes was explained by presenting the proportion of various risk factors that contributed to the overall risk in a pair of bar charts (Figure 2b), explicitly identifying contribution of familial risk (genetic predisposition).

Instead of the simple family history enquiry, participants in the intervention group completed a detailed and systematic family history questionnaire [25]. First participants had to indicate their number of children and siblings, and the number of both paternal or maternal aunts and uncles. Subsequently, they could indicate for each first and second-degree relative whether they were diagnosed with diabetes or whether they did not know this.

Besides the Diabetes Risk Test result, participants with a family history also received feedback about the total number of affected relatives based on their family history assessment. The bar chart (Figure 2b) was presented a second time.

### Outcome measures

Online questionnaires were administered at baseline, immediately post-test and after three months. In the control arm, the 3-month assessment included the detailed questionnaire to assess family history in this group.

#### Individuals with family history (research question 1)

**Primary outcomes***Saturated fat intake* was assessed with a validated food frequency questionnaire (FFQ) of 35-items, which measured the intake of food products that contribute most to saturated fat intake in the Netherlands [26]. A score for saturated fat intake, ranging from 0 to 80, was computed. The saturated fat score was not classified into categories, but was handled as a continuous variable, since an increase or decrease of 1 fat point is already an important change as has been shown by Oenema et al. [24]. Physical activity was assessed using the short version of the International Physical Activity Questionnaire (IPAQ) that measures frequency and duration of physical activity in the past 7 days [27]. According to the IPAQ data processing guidelines, participants were classified in a categorical score of three levels of physical activity: low, moderate, and high. Additionally participants were asked to indicate how many days a week they were physically active altogether for at least 30 minutes with, e.g. walking in fast pace, cycling, severe housekeeping, heavy work, gardening or sports. Attitudes towards testing for diabetes were assessed by a statement with 3 attitude items: “I think that regular (e.g. yearly) testing for diabetes with a blood glucose test is…” (not important [1] – important [7]; a bad idea [1] – a good idea [7]; not self-evident [1] – self-evident [7]). The 3 attitude items were combined in a scale, as internal consistency between the items was good (Cronbach’s alpha (α) was 0.88).

##### Secondary outcomes

Illness and risk perceptions were assessed at baseline and immediately post-test. To assess *causal beliefs* participants were asked to indicate the extent to which they believed different items could be a cause of diabetes (definitely not [1] – definitely [5]). This was based on the revised form of the Illness Perception Questionnaire [28] and comprised five items: heredity (diabetes runs in the family), predisposition, physical activity, healthy diet, being overweight. *Personal control over developing diabetes* was assessed using a 3-items scale (α =0.67): “There is a lot I can do to prevent getting diabetes”, “There is nothing I can do to decrease my risk of getting diabetes” (reversed), “I am definitely able to influence my risk of getting diabetes” (completely disagree [1] – completely agree [5]). *Perceived risk* was assessed by a single item: “In your opinion, what is the chance of you getting diabetes compared to an average man/woman your age?” (a lot lower [1] – a lot higher [7]). To assess *diabetes risk worry*, participants were asked to indicate their feelings when thinking about their chance of getting diabetes using a 7-point rating scale for two worry items (α =0.92) (no fear at all [1] – a lot of fear [7], not worried at all [1] - very worried [7]).

#### Individuals without family history (research question 2)

The primary outcome measure used to assess false reassurance was risk-reducing behaviour change and secondary outcome was perceived risk.

##### Socio-demographics

Socio-demographic variables (sex, age, ethnicity, educational level) were provided by the online research agency. Self-reported waist circumference, weight, and length were acquired from the baseline questionnaire.

### Sample size

In both research questions we hypothesized that there would be a change in risk-reducing behaviour. Sample size was calculated for all three risk-reducing behavioural outcome measures. The largest sample was required to demonstrate a change in fat intake, and thus this sample size is presented here (other sample size calculations see Additional file 2). Based on a significance level of 0.05 and a power of 0.80, the largest sample size for change in risk-reducing behaviour between baseline and 3-month follow-up was 291 subjects, based on a relative difference of 1.1 point on fat intake (range 0 – 80). The expectations of this effect (1.1) were based on results of a previous web-based trial [24].

### Statistical analyses

To identify predictors of participation and loss to follow-up, logistic regression analyses, with participation in trial (yes/no) and loss to follow-up (yes/no) as the dependent variable, were conducted. Independent variables were sex, age, education, BMI, and for loss to follow-up also baseline measures of physical activity level, fat intake, and attitudes towards testing for diabetes. Logistic regression analyses were conducted with study group as dependent variable to examine the similarity of the study groups at baseline. Independent variables for this analysis were sex, age, education, BMI, and follow-up measures of the detailed family history questionnaire. Chi-square tests were used to test for differences on the Diabetes Risk Test result between study groups. Linear regression analyses were conducted to test for follow-up group differences in outcome measures, with the follow-up measurement (3-month for behavioural outcomes and post-test for perceptions) of the outcomes as dependent variable, and study group and the baseline score of the outcome indicator as independent variables. Furthermore, we checked for effect modifiers in the analyses. Effect modification was defined as a significant (p<0.1) interaction term between the study group and variable of interest. In case of effect modification subgroup analyses were performed on the modifying variable. All analyses were performed as was intended according to the study protocol. The data were analyzed using SPSS version 18.0.

## Results

### Participants

Of the 1,300 participants randomly selected to participate in the web-based intervention, at 3-month follow-up 1,120 completed the questionnaire (86% response rate) (Figure 1). Response analyses showed no differences between participants and those who refused participation. No differences were found between individuals who were lost to follow-up after three months and respondents who completed all questionnaires (see Additional file 3: Table S3 and Additional file 4: Table S4). Baseline characteristics of the participants are shown in Table 1. In both cohorts of participants (with and without family history) there were no differences between the intervention and control group on these measures, as well as on baseline measures of the behavioural outcomes.

**Table 1:** Characteristics of participants

	**With family history**		**Without family history**	
	**Control**	**Intervention**	**Control**	**Intervention**
	**n=286**	**n=288**	**n=269**	**n=266**
Sex (% female)	54.2	55.2	43.9	48.9
Age (years, mean ± SD)	53.2 ± 5.9	53.5 ± 5.8	53.5 ± 5.3	53.4 ± 5.8
Ethnicity (% native Dutch origin)	97.2	95.8	97.8	98.5
Education^*^ (%)				
low	36.0	31.9	28.1	27.1
middle	41.3	44.1	47.9	44.0
high	22.4	24.0	24.0	28.9
BMI (%)				
overweight 25–29.9 kg/m^2^	67.1	64.9	66.9	67.7
obese ≥30 kg/m^2^	32.9	35.1	33.1	32.3
Familial risk for diabetes^†^				
average	-	-	96.3	92.9
moderate	66.1	67.0	1.5	4.5
high	33.9	33.0	2.2	2.6

The number of participants is those who were analyzed for the study.

^*^Low education refers to people who finished elementary school, lower secondary education or lower vocational education; Middle education refers to higher secondary education or intermediate vocational education; High education refers to university or higher vocational education.

^†^As assessed with the detailed family history questionnaire, high familial risk for diabetes refers to at least 2 affected first-degree relatives or at least 3 affected maternal or paternal relatives from the same lineage; moderate refers to 1 affected first degree relative, or 2 affected maternal or paternal second degree relatives from the same lineage; average refers to all others [43].

### Individuals with family history

Table 2 shows the scores on behavioural outcomes and illness and risk perceptions for baseline and the follow-up measurements for individuals with and without a family history and the outcomes of the regression analyses. Familial risk communication had no effect on saturated fat intake, physical activity level, or attitudes towards testing for diabetes. Education level was an effect modifier for the effect on saturated fat intake, therefore subgroup analyses were performed. A decrease in self-reported saturated fat intake for low-educated individuals in the intervention group when compared to the control group was found (Beta (*b*) -1.01, 95% confidence interval −2.01 to 0.00), whereas there was no effect for middle (*b*-0.37 (−0.51 to 1.25) and high educated individuals (*b −*0.61,-1.66 to 0.44). There was no effect of familial risk communication on causal beliefs, perceived personal control, and risk perception. However, there was a lower increase in worries about diabetes risk for individuals in the intervention group (*b −*0.21,-0.40 to −0.03). Table 3 shows that as a consequence of using a detailed familial risk assessment a greater proportion of individuals in the intervention group (96.2%) had a risk of 20 in 100 (highest diabetes risk result) compared to those in the control group (85.7%) within the cohort of individuals with a family history.

**Table 2:** Outcomes at baseline and follow-up and regression coefficients (b) for regression analyses

	**With family history**					**Without family history**				
	**Control**		**Intervention**			**Control**		**Intervention**		
	n=286^*^		n=288^*^			n=269^*^		n=266^*^		
	**Baseline**	**Follow-up**^**†**^	**Baseline**	**Follow-up**^**†**^	**b**^**‡ **^**(95%CI)**	**Baseline**	**Follow-up**^**†**^	**Baseline**	**Follow-up**^**†**^	**b**^**‡ **^**(95%CI)**
**Illness and risk perceptions**										
Causal beliefs (1–5)										
heredity	4.0 (0.9)	4.0 (0.7)	4.1 (0.8)	4.1 (0.8)	0.05 (−0.08 to 0.17)	3.8 (0.9)	3.9 (0.8)	3.8 (1.0)	4.0 (0.8)	**0.19 (0.06 to 0.33)**
predisposition	3.7 (0.8)	3.9 (0.7)	3.7 (0.8)	3.9 (0.8)	0.01 (−0.13 to 0.11)	3.6 (0.8)	3.7 (0.8)	3.6 (0.8)	4.0 (0.7)	**0.31 (0.19 to 0.43)**
physical activity	3.7 (0.9)	4.1 (0.7)	3.7 (0.9)	4.1 (0.7)	0.00 (−0.11 to 0.11)	3.6 (0.9)	4.0 (0.8)	3.6 (0.9)	4.1 (0.6)	0.04 (−0.07 to 0.16)
healthy diet	3.9 (0.8)	4.1 (0.7)	3.8 (0.8)	4.1 (0.8)	−0.02 (−0.13 to 0.10)	3.9 (0.8)	4.1 (0.7)	3.8 (0.9)	4.1 (0.6)	0.05 (−0.06 to 0.16)
overweight	4.2 (0.7)	4.2 (0.6)	4.2 (0.7)	4.2 (0.7)	−0.04 (−0.14 to 0.06)	4.2 (0.6)	4.2 (0.7)	4.1 (0.7)	4.2 (0.6)	0.02 (−0.08 to 0.13)
Personal control (1–5)	3.8 (0.6)	3.9 (0.6)	3.9 (0.5)	3.9 (0.5)	0.01 (−0.07 to 0.09)	3.8 (0.6)	4.0 (0.5)	3.8 (0.6)	3.9 (0.5)	−0.01 (−0.09 to 0.07)
Perceived risk (1–7)	4.5 (1.1)	4.7 (1.2)	4.5 (1.1)	4.8 (1.1)	0.12 (−0.03 to 0.27)	4.0 (1.1)	4.1 (1.2)	3.9 (1.1)	3.9 (1.2)	−0.12 (−0.29 to 0.04)
Diabetes risk worry (1–7)	3.5 (1.4)	3.9 (1.4)	3.6 (1.3)	3.8 (1.4)	**−0.21 (−0.40 to −0.03)**	3.3 (1.4)	3.6 (1.4)	3.1 (1.4)	3.4 (1.5)	−0.08 (−0.28 to 0.13)
**Behavioural outcomes**										
Sum score Fat list (0–80)	15.7 (5.2)	15.0 (5.3)	15.1 (5.3)	14.2 (5.5)	−0.29 (−0.85 to 0.27)	15.8 (5.0)	15.3 (5.1)	15.5 (5.2)	14.6 (4.9)	−0.49 (−1.00 to 0.05)
IPAQ categories^§^										
vigorous	57.7	58.4	51.0	56.3	0.03 (−0.08 to 0.13)	56.5	52.0	56.1	50,7	0.06 (−0.05 to 0.18)
medium	25.2	26.9	29.9	23.6		24.5	26.8	28.4	27,3	
low	10.5	10.1	13.5	14.2		13.8	15.6	11.2	11,9	
Days/wk physical active	4.3 (2.4)	4.1 (2.4)	4.2 (2.4)	4.2 (2.4)	0.20 (−0.15 to 0.54)	4.2 (2.4)	4.1 (2.5)	4.2 (2.4)	4.2 (2.4)	0.12 (−0.25 to 0.49)
Attitudes towards										
diabetes testing (1–7)	5.3 (1.3)	5.2 (1.3)	5.3 (1.3)	5.2 (1.3)	−0.07 (−0.23 to 0.09)	4.9 (1.4)	4.9 (1.4)	4.8 (1.4)	4.9 (1.3)	−0.03 (−0.20 to 0.13)

Data are means ± SD or percentages.

^*^The numbers are based on the availability of the detailed questionnaire to assess family history.

^†^Follow-up for illness and risk perception is directly post-test. For behavioural outcomes after three months.

^‡^The interpretation of the regression coefficient (b) for e.g. the illness perception diabetes risk worry (−0.21) would be that an individual in the intervention group will score 0.21 less on the perception of worries about their diabetes risk compared to an individual in the control group, when baseline values for diabetes risk would be similar.

^§^About 6% of the participants is missing due to data cleaning according the IPAQ data processing guidelines.

**Table 3:** **Diabetes risk presented to the participants based on the diabetes risk test**[23]

	**With family history**		**Without family history**	
	**Control**	**Intervention**	**Control**	**Intervention**
	n=286	n=288	n=269	n=266
Diabetes Risk Test result^*^	%	%	%	%
2 in 100	3.8	0	16.7	13.9
10 in 100	10.5	3.8	45.7	40.6
20 in 100	85.7	96.2	37.5	45.5
χ^2^-statistic; p-value^†^		21.8; p<0.001		3.5 p=0.17

^*^Indicating people’s risk of getting diabetes within the next five years.

^†^P-value based on the Chi-square test.

### Individuals without family history

Specifying the impact of familial risk of diabetes had no significant effect of the intervention on self-reported fat intake (*b −*0.49, -1.00 to 0.05), physical activity (*b* 0.06, -0.05 to 0.18), or attitudes towards diabetes testing (*b −*0.03, -0.20 to 0.13) in the none-family history group (Table 2). There was also no significant effect on perceived risk of diabetes in this group of participants (*b −*0.12, -0.29 to 0.04). In the intervention group there were higher scores for the perception that heredity (*b* 0.19, 0.06 to 0.33) and predisposition (*b* 0.31, 0.19 to 0.43) are important causes of diabetes. There was no effect of familial risk communication on other illness perceptions.

## Discussion

Overall, the addition of family history information to the web-based general diabetic risk information did not result in improvements in risk behaviour among participants with a diabetes family history. However, there are promising results for low-educated individuals, they were more likely to reduce their saturated fat intake. Also, the information reduced worry related to diabetic risk among participants with a family history. For individuals without relevant family histories, the emphasis on familial risk information did not result in false reassurance as demonstrated by no significant difference in risk-reducing behaviour and diabetes risk perception. Furthermore, a detailed family history assessment resulted in a greater percentage of individuals at familial risk for diabetes compared to a simple enquiry.

### Individuals with a family history

In contrast to findings in this study, an observational study and two controlled trials, that examined the impact of informing people about their familial risk for type 2 diabetes, reported increased self-reported risk-reducing behaviour [14,29,30]. These studies, however, involved a consultation with a health care professional. Perhaps, in that setting, the professional providing the information could verify whether people understood the complex interaction of genetic and behavioural causes and was able to give additional feedback and support. Moreover, individualized messages tailored to specific characteristics and knowledge of individuals about familial risk information personalized the risk, thus may be more persuasive than general healthy lifestyle advice, e.g. eat healthy [6]. It has been previously described that computer tailoring, as was performed in this study, lack features to imitate all characteristics of personal contact, since it mostly does not allow direct interaction between the respondent and the education expert [31]. Further, people might be more familiar with face-to-face contact. It was demonstrated in a study of Ziegellmann et al. [32] that middle aged individuals (40–54 years) benefit more from an interviewer assisted consultation, than a self-administered physical activity promoting intervention.

Lower socioeconomic groups often engage more in unhealthy behaviours [33], whilst interventions to improve health behaviours may result in greater uptake of the message in higher socioeconomic groups, resulting in an rising social inequality in health [34]. This population-wide study suggests an effect of familial risk information on saturated fat intake for low-educated individuals. This is consistent with another population-wide community-based health education intervention showing an improvement in fat intake among a low-educated population [35]. Further, low-educated individuals were more positive towards tailored dietary fat-feedback in earlier studies as compared to higher educated individuals [36]. Also, it was shown that overweight individuals are more likely to revisit a website, possibly because of the non-stigmatizing way of addressing body weight through the internet [37].

The incidental finding that 10% more individuals were classified at high diabetes risk (20 in 100 for getting diabetes within five years) in the intervention arm, suggests that incorporating a detailed family history questionnaire into the web-based assessment tool enhanced identification of individuals at high risk due to more accurate ascertainment of familial risk. This is supported by a related study [26] and has also been shown among persons with high cardiovascular risk in primary care practice [38].Currently the number of (online) self-administered risk assessment tools for common diseases is increasing. These tools often limit family history enquiry to a single question [23,39]. Integrating a detailed family history questionnaire to these risk assessments might result in a higher number of participants that will be identified as having a high diabetes risk, however this may take more effort.

A high baseline score in individuals with a family history on the questions enquiring about the perception that a cause of diabetes is heredity and predisposition left little room for improvement. This finding might indicate that individuals with a family history are already aware of family history as a risk factor. This is in line with earlier findings, that individuals with a family history indicated a parental history as the most important risk factor for diabetes [40]. Conversely, in this study, individuals without a family history were less aware of heredity and predisposition as a cause of diabetes at baseline, but their perception increased post-intervention.

Previous studies indicate that advising participants of their familial risk does not lead to sustained psychological harm [13-15]. In this study, those receiving general information had more worries than those receiving additional information related to familial risk. This suggests that explaining the role of familial risk raises understanding of the risk and moderates worries about diabetes risk.

### Individuals without a family history

It has been shown that a (favourable) negative test result on a screening for having type 2 diabetes does not lead to false reassurance, as demonstrated by no decrease in perceived diabetes risk, behavioural intentions, and self-rated health [20]. It might be anticipated if participants have no family history of diabetes, giving information about the significance of familial risk will lead to a decline in these participants’ perceived diabetes risk and adherence to risk-reducing behaviour [19]. However, there was no such false reassurance in this study, as indicated by no effect on risk perception and risk-reducing behaviour by participants without family history of diabetes.

Strength of this trial was that the participants were not aware of being selected on their diabetic family history, as this attribute was identified before the study. Further, there is no item non-response, since participants are obliged to give an answer to each question in order to complete the web-based questionnaire. A further strength is that the random sample was representative of the Dutch population with respect to sex and education, as these numbers were compared with data about the Dutch population [41]. However, the study population did not represent the ethnic mix of the Dutch population and reflected individuals with a BMI ≥25, therefore one should be cautious when generalizing these findings to the broader community. As the Diabetes Risk Test, used in this study, did not accurately predict the diabetes risk of non-Caucasian individuals, these minority populations were excluded from the study. Another limitation of the study is that self-reported measures were used for the behavioural outcomes; nevertheless all such measures were validated instruments. Objective measures, such as data from biomarkers or accelerometers were not feasible in this online research panel.

## Conclusions

Although there was generally no clear improvement in risk-reducing behaviour, the intervention improved dietary fat intake among lower educated participants. Often interventions to improve health behaviours widen the social inequality in health, i.e. low-educated individuals show more unhealthy behaviour [34]. However, this low-cost diabetes prevention tool with integrated familial risk information did show improved health behaviour among the subgroup of individuals at greatest need of preventative care. Conversely, a drawback of using web-based information is that it might lead to a selected utilisation. Younger, high-educated, employed, and healthier people have better access to internet and are more interested in health risk information [42]. The challenge is now to get individuals who would benefit the most to complete a diabetes prevention tool on the internet. Reassuringly, the incorporation of familial risk appears to reduce worry related to diabetic risk assessment. This would suggest a benefit from including familial risk identification in chronic disease assessment. Furthermore, incorporating familial risk identification into a diabetes prevention program does not lead to false reassurance in individuals identified without relevant family histories. In terms of identifying individuals at high diabetes risk, a detailed family history questionnaire identifies more individuals at familial risk than a simple enquiry and can contribute to a more correct familial risk identification in chronic disease assessment.

## Competing interests

No conflict of interest was reported by the authors of this paper.

## Authors’ contributions

MW researched data and wrote the manuscript. LH, NQ, PJK, MCC, DRMT contributed to the discussion and reviewed/edited the manuscript. MW and LH are taking responsibility for the contents of the article. All authors read and approved the final manuscript.

## Pre-publication history

The pre-publication history for this paper can be accessed here:

http://www.biomedcentral.com/1471-2458/13/485/prepub

## Supplementary Material

Additional file 1: Table S1 Differences in provided familial risk assessment information between the control and intervention group. Click here for file

Additional file 2 Sample sizes. Click here for file

Additional file 3: Table S3 Participation analysis. Click here for file

Additional file 4: Table S4 Non-response analysis. Click here for file
