# Supplementary material for: Using web-based familial risk information for diabetes prevention: a randomized controlled trial
Source: BMC Public Health. 2013 May 17;13:485. doi: 10.1186/1471-2458-13-485 (PMC3711930; doi:10.1186/1471-2458-13-485)
Supplement: Additional file 1: Table S1 — Differences in provided familial risk assessment information between the control and intervention group. [file 1471-2458-13-485-S1.doc]

**Supplemental Table S1 Differences in provided familial risk assessment information between the control and intervention group**

|  | Control group | Intervention group |
| --- | --- | --- |
| Pre-assessment information | - main risk factors (not including family history)  - effectiveness of preventive options | - main risk factors, emphasising family history (e.g. explaining that familial risk increases with the number and kinship of affected relatives)  - effectiveness of preventive options |
| Family history assessment | *Simple enquiry*: Participants were asked “Does diabetes occur within your family?”  1) no;  2) yes, with my grandfather, grandmother, uncle, aunt, nephew, niece;  3) yes, with my father, mother, brother, sister, or child.” | *Detailed questionnaire*: First, participants had to indicate the number of children and siblings, and the number of both paternal or maternal aunts and uncles. Subsequently, they could indicate for each first-degree relative and second-degree relative and whether these relatives had been diagnosed with diabetes or whether they did not know this. |
| Feedback information | - Individual risk based on the risk test  - Risk-reducing preventive measures | - Individual risk based on the risk test  - Information about the total number of affected relatives  - Risk-reducing preventive measures |
