# Supplementary material for: Using web-based familial risk information for diabetes prevention: a randomized controlled trial
Source: BMC Public Health. 2013 May 17;13:485. doi: 10.1186/1471-2458-13-485 (PMC3711930; doi:10.1186/1471-2458-13-485)
Supplement: Additional file 2 — Sample sizes. [file 1471-2458-13-485-S2.doc]

**Supplemental File S2 Sample sizes**

*Physical activity*: Based on a significance level of 0.05 and a power of 0.80, the minimum sample size of response for individuals with a family history at 3 months should be 284 subjects, to detect a relative difference of 0.2 on self-rated physical activity level (scale 1 – 5). The expectations of this effect were based on results of a previous web-based trial [24].

*Testing for diabetes*: Based on a significance level of 0.05 and a power of 0.80, the minimum sample size of response for individuals with a family history at 3 months should be 162 subjects, to detect a relative difference of 15% on testing for diabetes (Yes/No). The expectations of this effect were based on results of a previous study [14].
