# Supplementary material for: Using web-based familial risk information for diabetes prevention: a randomized controlled trial
Source: BMC Public Health. 2013 May 17;13:485. doi: 10.1186/1471-2458-13-485 (PMC3711930; doi:10.1186/1471-2458-13-485)
Supplement: Additional file 3: Table S3 — Participation analysis. [file 1471-2458-13-485-S3.doc]

**Supplemental Table S3 Participation analysis**

|  |  | Participants | Decliners for participation | p-value* |
| --- | --- | --- | --- | --- |
|  |  | (n=1863) | (n=145) |  |
| Sex (% female) | | 46.2 | 50.1 | 0.17 |
| Age (years, mean ± SD) | | 53.5 (5.7) | 53.3 (5.5) | 0.71 |
| Education^†^ (%) | |  |  |  |
|  | low | 35.9 | 30.9 |  |
|  | middle | 42.8 | 43.9 | 0.08 |
|  | high | 20.0 | 24.9 |  |
| BMI (%) | |  |  |  |
|  | overweight 25-29.9 kg/m^2^ | 66.5 | 63.4 | 0.46 |
|  | obese ≥30 kg/m^2^ | 33.5 | 36.6 |  |

* p-values are based on logistic regression analyses.

^†^ Low education refers to people who finished elementary school, lower secondary education or lower vocational education; Middle education refers to higher secondary education or intermediate vocational education; High education refers to university or higher vocational education.
