# Supplementary material for: Using web-based familial risk information for diabetes prevention: a randomized controlled trial
Source: BMC Public Health. 2013 May 17;13:485. doi: 10.1186/1471-2458-13-485 (PMC3711930; doi:10.1186/1471-2458-13-485)
Supplement: Additional file 4: Table S4 — Non-response analysis. [file 1471-2458-13-485-S4.doc]

**Supplemental Table S4 Non-response analysis**

|  |  | Responders | Non-responders | p-value* |
| --- | --- | --- | --- | --- |
|  |  | (n=1109) | (n=54) |  |
| Sex (% female) | | 50.7 | 44.4 | 0.94 |
| Age (years, mean ± SD) | | 53.4 (5.7) | 54.7 (5.6) | 0.08 |
| Education^†^ (%) | |  |  |  |
|  | low | 30.8 | 25.9 | 0.93 |
|  | middle | 44.2 | 53.7 |  |
|  | high | 24.7 | 20.4 |  |
| BMI (%) | |  |  |  |
|  | overweight 25-29.9 kg/m^2^ | 66.6 | 75.9 | 0.34 |
|  | obese ≥30 kg/m^2^ | 33.4 | 24.1 |  |
| Sum score Fat list (0-80) | | 15.5 | 15.7 | 0.97 |
| IPAQ categories^‡^ | |  |  |  |
|  | vigorous | 16.7 | 12.4 | 0.27 |
|  | medium | 25.9 | 27.0 |  |
|  | low | 51.9 | 55.3 |  |
| Days/wk physical active | | 4.2 | 4.4 | 0.37 |
| Attitudes towards diabetes testing (1-7) | | 5.1 | 5.0 | 0.46 |

* p-values are based on logistic regression analyses.

^†^Low education refers to people who finished elementary school, lower secondary education or lower vocational education; Middle education refers to higher secondary education or intermediate vocational education; High education refers to university or higher vocational education.

^‡^About 6% of the participants is missing due to data cleaning according the IPAQ data processing guidelines.
